# Frmpd1 Facilitates Trafficking of G-Protein Transducin and Modulates Synaptic Function in Rod Photoreceptors of Mammalian Retina

**DOI:** 10.1523/ENEURO.0348-22.2022

**Published:** 2022-10-14

**Authors:** Christie K. Campla, Ulisse Bocchero, Ryan Strickland, Jacob Nellissery, Jayshree Advani, Irina Ignatova, Dhiraj Srivastava, Angel M. Aponte, Yuchen Wang, Jessica Gumerson, Kirill Martemyanov, Nikolai O. Artemyev, Johan Pahlberg, Anand Swaroop

**Affiliations:** 1 Neurobiology, Neurodegeneration and Repair Laboratory, National Eye Institute, National Institutes of Health, Bethesda, MD 20892; 2 Photoreceptor Physiology Group, National Eye Institute, National Institutes of Health, Bethesda, MD 20892; 3 Department of Molecular Physiology and Biophysics, University of Iowa Carver College of Medicine, Iowa City, IA 52242; 4 Proteomics Core, National Heart Lung and Blood Institute, National Institutes of Health, Bethesda, MD 20892; 5 Department of Neuroscience, The Scripps Research Institute, Jupiter, FL 33458; 6 Department of Ophthalmology and Visual Sciences, University of Iowa Carver College of Medicine, Iowa City, IA 52242

**Keywords:** G-protein, phototransduction, rod photoreceptor, rod-rod bipolar signaling, transducin translocation, vision

## Abstract

Trafficking of transducin (Gα_t_) in rod photoreceptors is critical for adaptive and modulatory responses of the retina to varying light intensities. In addition to fine-tuning phototransduction gain in rod outer segments (OSs), light-induced translocation of Gα_t_ to the rod synapse enhances rod to rod bipolar synaptic transmission. Here, we show that the rod-specific loss of Frmpd1 (FERM and PDZ domain containing 1), in the retina of both female and male mice, results in delayed return of Gα_t_ from the synapse back to outer segments in the dark, compromising the capacity of rods to recover from light adaptation. Frmpd1 directly interacts with Gpsm2 (G-protein signaling modulator 2), and the two proteins are required for appropriate sensitization of rod-rod bipolar signaling under saturating light conditions. These studies provide insight into how the trafficking and function of Gα_t_ is modulated to optimize the photoresponse and synaptic transmission of rod photoreceptors in a light-dependent manner.

## Significance Statement

Light-dependent trafficking of G-protein transducin (Gα_t_) in rod photoreceptors is critical for extending the range of light intensities at which rod-mediated vision can take place. Here, we demonstrate that a rod-specific isoform of Frmpd1 (FERM and PDZ domain containing 1) modulates retrograde transport of Gα_t_ during dark adaptation through its interactions with Gpsm2 (G-protein signaling modulator 2). In addition, both Frmpd1 and Gpsm2 are required for optimization of rod to rod-bipolar synaptic transmission when Gα_t_ is present at the synapse. Our studies provide a unifying link between Gα_t_ trafficking and function with broad implications for understanding the adaptive response of rod photoreceptors.

## Introduction

Rod and cone photoreceptors in the retina serve as the point of photon capture, converting light stimuli into electrical signals processed by inner retinal neurons before transmission to the rest of the brain. Both rods and cones use similar mechanisms for phototransduction, yet possess key morphologic, molecular, and physiological differences conferring the ability to respond to specific wavelengths and intensities of light (Kefalov et al., 2003; Lamb, 2013; Ingram et al., 2016). In rod outer segments (OS), G-protein coupled receptor rhodopsin can be activated by a single photon (Baylor et al., 1979; Pugh, 2018), in turn activating multiple G-protein α subunits (rod transducin Gnat1, here referred to as Gα_t_) on the OS disk membrane (Yue et al., 2019). Exchange of GDP for cytosolic GTP results in dissociation of the Gα_t_ subunit from Gβγ_T_ to interact with phosphodiesterase (PDE), which hydrolyzes intracellular cGMP (Arshavsky and Burns, 2012; Gulati and Palczewski, 2021). Reduced cGMP concentration leads to cation selective cGMP-gated channel closure and membrane hyperpolarization (Arshavsky and Burns, 2012). Consequent reduction in glutamate release at the rod synapse allows for graded signaling to downstream bipolar and horizontal cells (Witkovsky et al., 1997).

In darkness, Gα_t_ is most concentrated in rod OS, bound to disk membranes (Slepak and Hurley, 2008). Following extended exposure to bright light, activated Gα_t_^GTP^ and Gβγ_T_ dissociate from disk membranes and translocate to inner segments (IS) and synapses (Sokolov et al., 2002; Artemyev, 2008; Tian et al., 2013). This translocation is suggested to reduce phototransduction gain and facilitate light adaptation of the rod photoresponse (Sokolov et al., 2002; Calvert et al., 2006; Frederiksen et al., 2021), in addition to lowering the stress and metabolic demand induced by constitutive activation of rod phototransduction under bright light illumination (Elias et al., 2004; Fain, 2006; Peng et al., 2011; Majumder et al., 2013; Tian et al., 2014). Moreover, Gα_t_ translocation to the rod synapse has been shown to modulate synaptic signaling by enhancing rod transmission to rod bipolar cells (RBCs) under bright light conditions (Majumder et al., 2013). Upon return to a dark state, Gα_t_^GTP^ is hydrolyzed to Gα_t_^GDP^, recombines with Gβγ_T_ and returns to the OS (Artemyev, 2008; Frederick et al., 2020). This bi-directional translocation has been hypothesized to involve passive diffusion mediated by binding site “sinks” (Nair et al., 2005b; Slepak and Hurley, 2008), but involvement of active transport is also suggested (Peterson et al., 2005; Reidel et al., 2006; Artemyev, 2008). Although massive light-induced translocation of Gα_t_ to IS and synapses is well established, mechanisms for how Gα_t_ returns to OS or modulates synaptic transmission remain unclear.

Recent studies uncovered protein interactors involved in the unique trafficking and function of Gα_t_ in rod IS and synapses (Frederick et al., 2020; Srivastava et al., 2020). Gpsm2 (G-protein signaling modulator 2, LGN) localizes to these subcellular compartments and stabilizes inactive, GDP-bound Gα_t_ *in vitro* (Kerov et al., 2005a; McCudden et al., 2005; Nair et al., 2005a). We identified a unique transcript of *Frmpd1* (FERM and PDZ domain containing 1) derived from an alternate promoter and specifically expressed in rods and RBCs (Campla et al., 2019). Reported Frmpd1-Gpsm2 interactions (Pan et al., 2013) suggest an important role of Frmpd1 in rod function. Curiously, Frmpd4 has been linked to mGluR regulation and dendritic spine morphogenesis (Hu et al., 2017; Piard et al., 2018), whereas Frmpd2 interacts with Lrit1 in cone photoreceptors to modulate synaptic transmission to ON-bipolar cells (Sarria et al., 2018; Ueno et al., 2018).

Here, we provide molecular and electrophysiological evidence that Frmpd1 accelerates trafficking of Gα_t_ back to rod OS following light-induced translocation. Furthermore, our data indicate an important functional role for Frmpd1 and Gpsm2 in enhancing rod to RBC synaptic transmission in bright light conditions. Taken together, these data offer a plausible unifying mechanism to explain how trafficking and function of Gα_t_ are interrelated and dependent on cell compartment-specific interactions coordinated by Frmpd1.

## Materials and Methods

### Mouse lines and animal husbandry

All experiments were conducted according to protocols approved by a local Institutional Animal Care and Use Committee (ASP650) and adhered to the Association for Research in Vision and Ophthalmology statement for animal use in ophthalmic and vision research. *Frmpd1^Δ1a^* mice were generated as previously described (Campla et al., 2019), and Gpsm2^−/−^ mice (Tarchini et al., 2013) were graciously provided by M. Cayouette.

### Experimental design and statistical analysis

Relevant experimental design and statistical analysis details are outlined in each section below. Male and female mice were used in roughly equal proportions in each experiment, comparing littermates of genotypes whenever possible, and a minimum of three biological replicates were performed for all experiments. Data were prepared for analysis using GraphPad Prism software unless otherwise specified and presented as mean ± SEM (error bars) in figures and Table 1.

**Table 1.** Rod and rod bipolar cell (RBC) response properties

	I_1/2_ (R*/rod)	I_max_ (pA)	TTP (ms)	*N*
DA Rods				
Wildtype	15.1 ± 1.3	21.7 ± 2.3	162 ± 10	13
Frmpd1^Δ1^*^a^*	20.5 ± 2.2	15.6 ± 1.2	164 ± 12	7
Gpsm2^-/-^	19.5 ± 1	23.4 ± 2.8	168 ± 7	7
TS Rods				
Wildtype	200.6 ± 1	2.7 ± 0.7	52 ± 3	7
Frmpd1^Δ1^*^a^*	177.6 ± 7.8	3.7 ± 0.4	63 ± 7	4
Gpsm2^-/-^	206 ± 14.6	2.3 ± 0.5	53 ± 8	3
DA RBCs				
Wildtype	3.6 ± 0.2	263 ± 24.4	142 ± 5	29
Frmpd1^Δ1^*^a^*	4.1 ± 0.3	254 ± 40	135 ± 3	14
Gpsm2^-/-^	3.2 ± 0.3	317 ± 55	139 ± 4	5
TS RBCs				
Wildtype	19 ± 3.4	181.2 ± 47.8	75 ± 3	5
Frmpd1^Δ1^*^a^*	57.3 ± 3.3***	93 ± 24.8	80 ± 3	4
Gpsm2^-/-^	48.7 ± 5.4**	89.3 ± 15.8	74 ± 3	5

Sensitivity (I_1/2_), maximum responses amplitude (I_max_), and time to peaks (TTP) of wild-type, *Frmpd1^Δ1a^*, and Gpsm2^−/−^ in both dark-adapted (DA) and translocated (TS) states. Data are shown as mean ± SEM, number of cells (*N*).

### Plasmid constructs

pUB-GFP was a gift from Connie Cepko (Addgene plasmid #11155; Matsuda and Cepko, 2004). The full-length protein coding region of murine Frmpd1 was PCR amplified from C57BL/6J mouse cDNA using a high-fidelity Taq polymerase (SeqAmp DNA polymerase, Clontech) with in-frame FLAG epitope on its C-terminus (forward primer: 5′-CGCAGGTACCATGGAAGAGCTGGACGGCAG-3′; reverse primer: 5′-TGCAGCGGCCGCTCACTTGTCGTCATCGTCTTTGTAGTCCAGAGCGGTGGACGCCCGG-3′) and inserted in place of GFP in pUB-GFP construct. pTK47-mCherry-LGN-hardened was obtained from Addgene (Ian Cheeseman Lab). This plasmid contains the human *gpsm2* gene fused to the C-terminus of m-Cherry in the vector backbone from pEGFP-C1. It encodes m-Cherry-Gpsm2 fusion protein of 95 kDa.

### *In vivo* electroporation

DNA solutions were prepared from concentrated plasmid DNA stocks and fast green tracking dye diluted to desired final concentrations (1.8 μg/μl FLAG-tagged Frmpd1, 1 μg/μl pUb-GFP for 1:1 molar ratio) in molecular grade water. Neonatal (P1) CD1 mouse pups were administered ketoprofen and anesthetized by placing on ice for several minutes, after which the right eyelid was sterilized with 70% ethanol. A cut was carefully made along the future eyelid furrow using a sterile 30-gauge needle to expose the eye, and a small puncture was made using a fresh sterile 30-gauge needle at the nasal side of the corneal-scleral junction. A blunt-ended syringe (Hamilton Company) was then carefully passed through the puncture and behind the lens to the subretinal space where 0.4 μl of DNA solution was slowly and uniformly injected. Electrical pulses (five pulses at 80 V, 50-ms pulse with 950-ms interval) were applied across the head using a BTX ECM 830 apparatus (BTX Harvard Bioscience) with tweezer electrodes (Tweezertrodes, BTX Harvard Bioscience), after which pups were placed on a heating pad until recovered.

### Light-induced Gα_t_ translocation

Mice were first placed in complete darkness overnight, serving as dark-adapted time point 12 h (DA). The following day, phenylephrine ophthalmic (1%), proparacaine HCl (0.5%), and tropicamide (1%) solution was administered to each eye. Mice were then placed in a light box (∼1000 lux) for 1.5 h, serving as light-adapted time point 0 h (LA). Mice were then returned to complete darkness for 2 h. All mice were sacrificed at the indicated time point via cervical dislocation, and eyes were immediately enucleated for dissection under dim red light (dark-adapted time points) or ambient lighting (light-adapted time points).

### Cryosection immunohistochemistry

**Figure 1.**
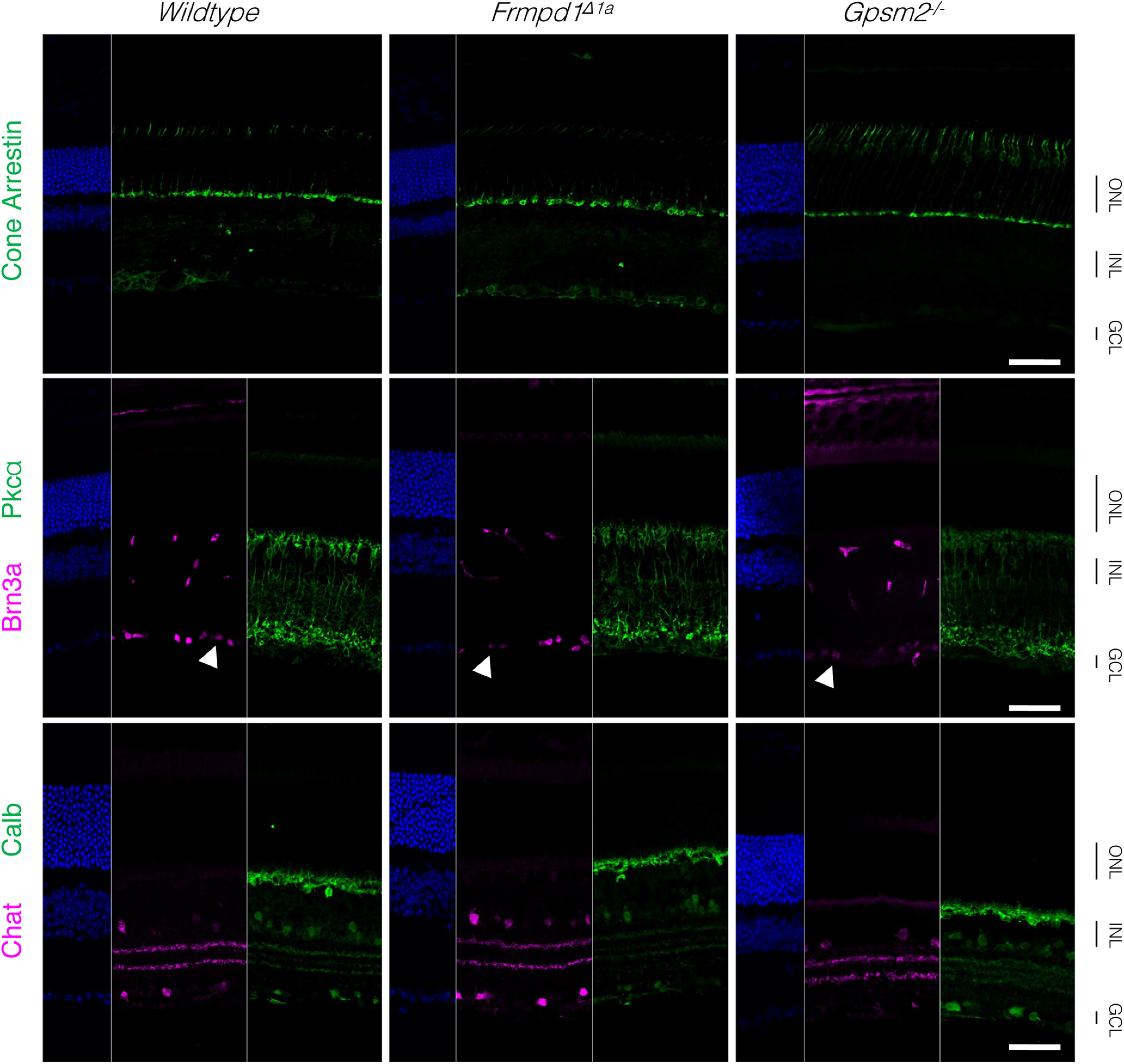
Gross retinal morphology is unaltered in *Frmpd1* and *Gpsm2*-knock-down mouse lines. Immunostaining of Post natal day 21 (P21) mouse retina sections was performed to assess various cell types. Primary antibodies were used to detect cones (Cone Arrestin), ganglion cells (Brn3a, arrows), bipolar cells (Pkcα), horizontal cells (Calbindin), and amacrine cells (Chat). Scale bar: 50 μm. ONL, outer nuclear layer; INL, inner nuclear layer; GCL, ganglion cell layer.

**Figure 2.**
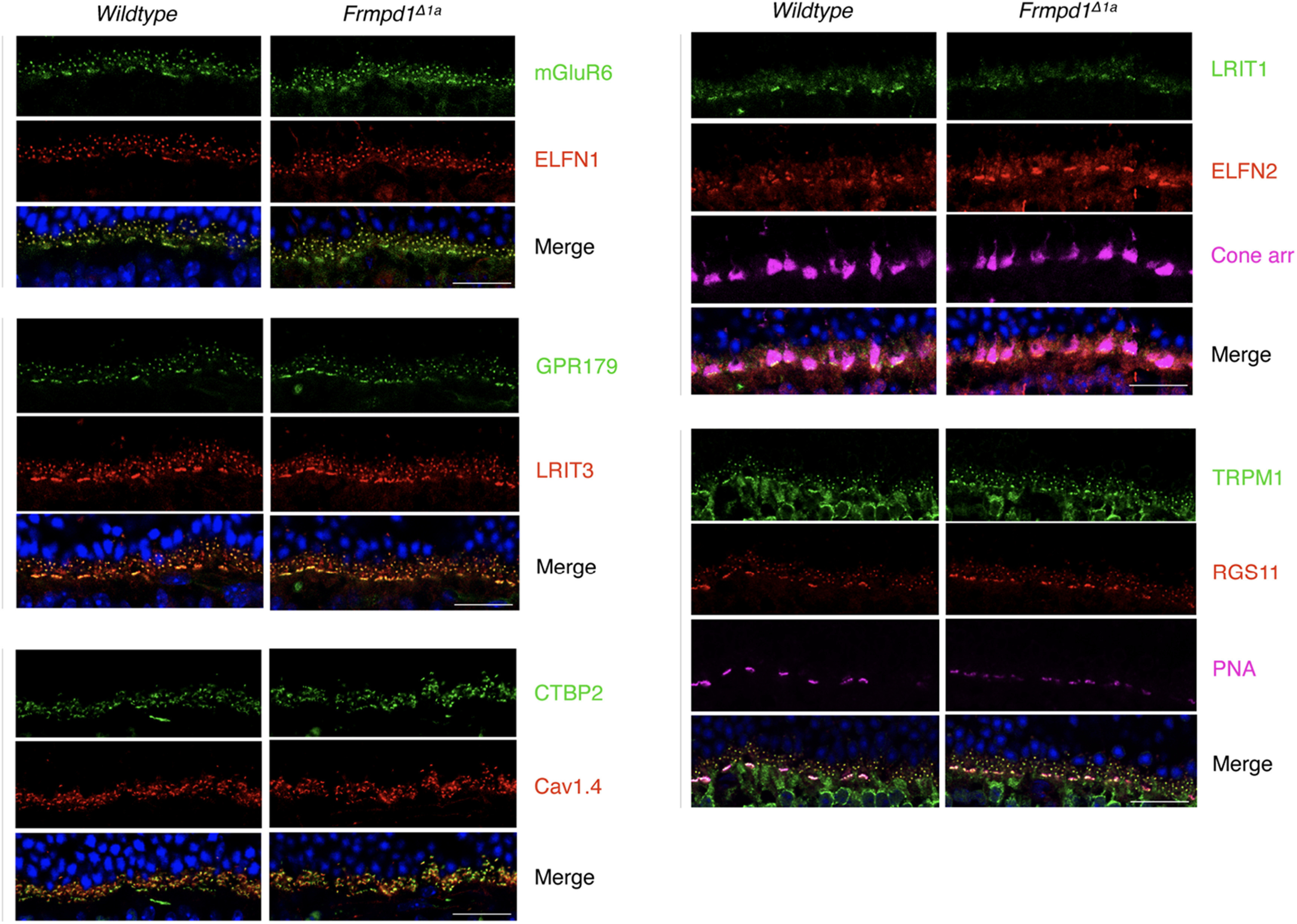
Outer plexiform layer (OPL) synaptic morphology is unaltered in *Frmpd1^Δ1a^* mice. Morphologies of OPL synapses were examined by immunostaining using frozen retina sections from Post natal day 21 (P21) mice. Scale bar: 20 μm.

Eyes were immersed in 4% PFA at room temperature for up to 2.5 h and the cornea, iris, and lens were subsequently removed to create an eyecup. Eyecups were cryoprotected in 30% sucrose in PBS overnight at 4°C before being embedded in Tissue-Tek OCT (Sakura). Frozen cryosections were blocked for 1 h at room temperature in blocking solution (5% normal donkey serum, 0.3% Triton X-100 dissolved in 1× PBS then passed through a 0.22-μm filter). Sections were incubated overnight in a humidified chamber at 4°C with primary antibody diluted in blocking solution. They were then washed with 0.1% Triton X-100 in 1× PBS at room temperature, and incubated at room temperature for 1 h with AlexaFluor-conjugated secondary antibody diluted 1:500 in blocking solution. Sections were again washed and stained for 5 min with DAPI in 1× PBS, washed again, then mounted with Fluoromount-G (Southern Biotech). Samples were imaged with a Zeiss 700 confocal microscope and processed with ImageJ software. Synaptic immunohistochemistry in Figures 1, 2 was performed similarly, as described (Wang et al., 2017; Sarria et al., 2018; Cao et al., 2020). Antibody details provided in Table 2.

**Table 2:** Antibodies used for immunofluorescence, Co-Immunoprecipitation and immunoblotting

Antibody	Host species	Source	Dilution		
			IHC	Immunoblot	Immuno-precipitation
Brn3a	Mouse	Santa Cruz; sc8429	1:1000	-	-
Calbindin	Rabbit	Calbiochem; PC253L	1:1000	-	-
Cav1.4	Rabbit	A. Lee (Liu et al., 2013)	1:1000	-	-
Chat	Goat	Millipore; AB144P	1:200	-	-
Cone arrestin	Rabbit	Millipore; AB15282	1:1000	-	-
Ctbp2	Mouse	BD Biosciences; 612044	1:1000	-	-
Elfn1	Rabbit	K. Martemyanov (Cao et al., 2015)	1:100	-	-
Elfn2	Rabbit	Thermo Fisher Scientific; PA5-43521	1:100	-	-
Flag	Rabbit	Cell Signaling; 2368	1:600	1:1000	-
Flag	Mouse	Sigma; F1804	1:200	-	3 μg/1600 μg lysate
Frmpd1	Rabbit	Atlas Antibodies; HPA042934	-	1:1000	3 μg/1600 μg lysate
Gpr179	Mouse	Primm Biotech; Ab887	1:250	-	-
Gpsm2	Rabbit	Millipore; ABT174	-	1:500	-
Gpsm2	Goat	AntibodiesOnline; ABIN190875	-	-	3 μg/1600 μg lysate
Lrit1	Mouse	Santa Cruz; sc-376508	1:100	-	-
Lrit3	Rabbit	C. Zeitz (Neuillé et al., 2015)	1:200	-	-
mGlur6	Sheep	K. Martemyanov (Cao et al., 2011)	1:200	-	-
Pkcα	Rabbit	Sigma; P4334	1:1000	-	-
Pmca	Mouse	Abcam; AB2825	1:200	-	-
PNA (AlexaFluor 647-conjugated)	n/a	Life Technologies; L32460	1:1000	-	-
RFP	Rabbit	Rockland Immunochem; 600-401-379	-	1:2000	3 μg/1600 μg lysate
Rgs11	Rabbit	K. Martemyanov (Cao et al., 2008)	1:500	-	-
Transducin, alpha subunit (Gα_t_)	Rabbit	Proteintech; 55167-1-AP	1:500	-	-
Transducin, alpha subunit (Gα_t_)	Rabbit	Santa Cruz; sc389	-	1:250	-
Trpm1	Sheep	K. Martemyanov (Cao et al., 2011)	1:500	-	-
β-Actin	Mouse	Sigma; A5316	-	1:3000	-

### Transducin quantification assay

Transducin-stained sections were imaged as confocal z-stacks with the observer blind to sample genotypes, and maximum intensity projection images were then generated and quantified using ImageJ. For each image, five equal-sized square regions of interest (ROIs) were selected from four different retinal layers (i.e., 20 ROI per image): outer segment, inner segment, synapse, or background (inner nuclear layer). Mean gray value was measured for each ROI, and the ROIs for each layer were averaged, generating four values for each image, one per layer. The background was subtracted, and total fluorescence (F_tot_) was estimated by summing the remaining three photoreceptor layers. The relative intensity of each layer was determined as a ratio of the total. Three separate experiments were performed and the relative intensities for each of the three photorecepter layers across all datasets were included for performing statistical analysis (*n* = 20–21 images from 3–4 mice per genotype). GraphPad Prism software was used to determine statistical significance with a one-way ANOVA and Dunnett’s multiple comparison test performed for each layer separately. Statistical significance is denoted with an asterisk where *p* < 0.05. Data are presented as mean ± SEM (error bars).

### Single-cell electrophysiological recordings

Light responses from rod photoreceptors and rod bipolar cells were recorded from retinal slices as previously described (Pahlberg et al., 2018; Beier et al., 2022). Briefly, slices were obtained from mice dark-adapted overnight and euthanized according to protocols and guidelines approved by the NIH. Mouse eyes were dissected under infrared light (Thorlabs 940-nm LED), and retinas were extracted and embedded in low density agar (3%) in HEPES-buffered Ames’ media (10 mm HEPES, pH 7.4). The retina was cut with a vibratome to obtain 200 μm thick slices. The quality of the preparation was assessed by visualizing each slice under infrared illumination. The selected slice for the experiment was superfused with Ames’ medium with an 8 ml/min flow rate, equilibrated with 5% CO_2_/95% O_2_, and maintained at 35–37°C. The internal solution for whole-cell patch-clamp recordings contained (in mm): 125 K-aspartate, 10 KCl, 10 HEPES, 5 *N*-methyl glucamine-HEDTA, 0.5 CaCl_2_, 1 ATP-Mg, and 0.2 GTP-Mg; pH was adjusted to 7.3 with NMG-OH. Light-evoked responses were measured using patch electrodes with a 12–15 MΩ resistance, in voltage-clamp mode (holding potential for rods and bipolar cells was −40 and −60 mV, respectively). Light responses were obtained by delivering 20-ms flashes from a blue-green LED (λmax ∼ 505 nm). Flash strengths varied from producing just-measurable responses, to those that produced a maximal response, increasing with a factor of two. Responses were low pass filtered at 300 Hz and sampled at 10 kHz.

Transducin (Gα_t_) translocation was achieved by subjecting mice to 800 lux light for 45 min. Pupils were completely dilated with a drop of a solution containing phenylephrine ophthalmic (1%), proparacaine HCl (0.5%) and tropicamide (1%) on the cornea. After the translocation protocol, mice were dark-adapted for 30 min to obtain some visual pigment regeneration and recovery of visual sensitivity. All experiments were halted 45–60 min after the start of recordings to ensure Gα_t_ remained substantially in the inner segment (Sokolov et al., 2002; Majumder et al., 2013). These data were combined and averaged for each experiment, and the obtained values were used to represent response properties for the different genotypes in their translocated state, respectively.

### Electroretinogram (ERG) recordings

Mice were dark-adapted overnight and anesthetized by intraperitoneal injection of ketamine (80 mg/kg) and xylazine (8 mg/kg). A second and occasionally third dose of anesthetic was given ∼30–40 min apart, to extend the duration of the experiments. Pupils were dilated as described for single cell recordings and 2.5% hypromellose ophthalmic demulcent solution was used to maintain moisture of the eyes. Mice were transferred to a heating pad (37°C) and ERG responses were recorded from both eyes using gold wire loop electrodes placed over each cornea. Dark-adapted ERG responses were obtained using 20-ms light flashes of increasing light intensities (0.0012 to 274 cd·s/m^2^). Responses were filtered at 5 kHz and sampled at 2 kHz. Each light response was averaged from three to four recordings every 10–90 s, depending on stimulus intensity. Following dark-adapted ERG recordings, mice were exposed to 800 lux light for 30–35 min to induce Gα_t_ translocation. Light responses were obtained using a 20-ms flashes of 36 cd·s/m^2^ with an intertrial interval of 60 s every ∼5 min until the end of anesthesia.

### Electrophysiological data analysis

Patch-clamp and ERG analysis was performed with a custom MATLAB software package. Light sensitivity for each mouse genotype and for each cell type was estimated from the half-maximal flash strength (I_1/2_) obtained from the best fit Hill equation to the normalized data. All electrophysiology figures were made using Igor 8.0 from Wavemetrics. Analysis code is available at https://github.com/PahlbergLab. Statistical significance was assessed with two-tailed unpaired Student’s *t* test with unequal variances and defined as *p < 0.05, **p < 0.01, ***p < 0.001. Data are reported as mean ± SEM, number of cells (*N*).

### Immunoprecipitation (IP)

Retinas were collected and snap-frozen after 1-h DA and stored at −80°C before IP (*n* = 10/group). Frozen retinas were resuspended in ice-cold IP buffer (150 mm NaCl, 3 mm EDTA, 40 mm Tris, 10% glycerol, 1% w/v n-dodecyl-β-D-maltoside, 1× Roche protease inhibitors, prepared fresh) on ice, homogenized with a handheld plastic pestle homogenizer, and sonicated. HEK293 lysates grown to confluency in six-well plates were similarly processed by lysing in 500-μl IP buffer. Supernatant was quantified by BCA assay and diluted to yield 1600 μg and an aliquot was saved as “input” fraction. A total of 3 μg of primary antibody (refer to Table 2 for antibody details) was added to each tube of retina lysate and mixed on a rotating wheel overnight 4°C. The next day, 50 μl (1.5 mg) of Dynabeads protein A (for rabbit antibodies) or protein G (for goat antibodies) were added and mixed on a rotating wheel at 4°C for 2 h to capture protein complexes. Beads were washed 3 × 10 min on a rotating wheel at 4°C in IP buffer, and protein complexes were released from beads by a denaturing elution in 45 μl IP buffer + 25 μl 4× Laemmli buffer (Bio-Rad) + 355 mm β-mercaptoethanol for 10 min at 105°C and separated by SDS-PAGE for immunoblotting.

### Immunoblotting

Supernatants were solubilized in 4× Laemmli buffer (Bio-Rad) + β-mercaptoethanol (355 mm final concentration) for 10 min at 105°C. After denaturation, protein extracts were separated by SDS-PAGE using Mini-PROTEAN TGX Precast Gels (Bio-Rad) and transferred to polyvinylidene (PVDF) membrane using preprogrammed high molecular weight protocol of TransBlot Turbo Transfer System (Bio-Rad). Membranes were blocked for 1 h at room temperature in Easyblock solution (Genetex), then incubated with primary antibody diluted in Easyblock overnight at 4°C. The next day, membranes were washed 4× for 10 min each with TBST at room temperature, then incubated for 1 h at room temperature with horseradish peroxidase (HRP)-conjugated Easyblot secondary antibody (Genetex) diluted in Easyblock solution. After four more TBST washes, membranes were developed with SuperSignal West Pico Chemiluminescent Substrate (Thermo) or SuperSignal West Femto Maximum Sensitivity Substrate (Thermo), then imaged on ChemiDoc Touch Gel Imaging System (Bio-Rad). For subsequent probing, membranes were stripped with a mild 0.2 m glycine solution (pH 2.2) for 4 min 50°C before being re-blocked and re-probed. Antibody details provided in Table 2.

### Bio-Layer Interferometry (BLI) binding assay

Gpsm2 was cloned into modified pET21a vector with N terminal His6-MBP tag and TEV protease cleavage site (HMT-Gpsm2) and transformed into *Escherichia coli* strain Rosetta 2 (DE3; Novagen) cells. Chimeric transducin-α-like Gαt, Chi8 (Gα_t_*; Skiba et al., 1996; Natochin et al., 1998) was cloned into modified pET21a with N terminal His6 tag and TEV protease cleavage site. Frmpd1 residue 895–938 was cloned into a modified pET21a vector with N terminus His6, avi and thioredoxin tag and TEV protease cleavage site (His-Avi-Trx-Frmpd1-pept). We used Frmpd1 residue 895–938 for the BLI and pull-down experiment since these residues are the only binding site for Gpsm2 in Frmpd1 identified in earlier studies (Pan et al., 2013). Also, expression and large-scale purification of the large and mostly unstructured full-length Frmpd1 for *in vitro* biochemical studies was not feasible. Plasmids containing Gα_t_* and His-Avi-Trx-Frmpd1-pept constructs were transformed into *E. coli* strain BL21 (DE3; Novagen). Cells expressing HMT-Gpsm2 and Gα_t_* were grown to OD_600_ = 0.6 in 2XTY medium at 37°C and induced with 50 μm IPTG at 18°C overnight. Cells expressing Frmpd1 construct was grown in LB media at 37°C to OD_600_ = 0.6 and 20 mg of D-biotin was added per liter of LB media before inducing with 100 μm IPTG. Cells were further grown overnight at 22°C overnight.

Cells expressing HMT-Gpsm2, Gα_t_*, and His-Avi-Trx-Frmpd1-pept were resuspended in buffer N1 (50 mm HEPES, 300 mm NaCl, 5% glycerol, pH 8.0) supplemented with a Complete, Mini, EDTA-free Protease Inhibitor Cocktail tablet (Roche) and 2 mm PMSF. For Gα_t_*, cell suspensions was also supplemented with 10 mm MgCl_2_ and 50 μm GDP. Cells were lysed by sonication, cell debris was cleared by centrifugation and supernatant was loaded onto His-bind resin (EMD Millipore) charged with Ni^++^. Resin was washed with five-column volumes of resuspension buffer followed by buffer N1 containing 30 mm imidazole. Proteins were eluted with buffer N1 containing 300 mm imidazole. HMT-Gpsm2 was dialyzed in 20 mm Tris buffer (pH 7.5) containing 5% glycerol, 25 mm NaCl, 5 mm β-mercaptoethanol (buffer S1) and further purified by SP-Sepharose (GE Healthcare) cation exchange chromatography. Resin was washed first with buffer S1 and then with buffer S1 containing 25 mm NaCl. Protein was eluted using buffer S1 containing 400 mm NaCl. Gα_t_* was dialyzed in to buffer S1 and further purified by anion exchange chromatography. Gα_t_* was loaded onto HiTrapQ column (GE Healthcare) and eluted with linear gradient of NaCl from 0.05 to 1 m NaCl. As the final step of purification, Gpsm2 was purified by size-exclusion chromatography (SEC) using HiLoad 16/600 Superdex 200 pg column equilibrated with 20 mm Tris, 150 mm KCl, 5% glycerol, 1 mm TCEP, pH 7.5. Gα_t_* and His-Avi-Trx-Frmpd1-pept was purified by SEC using HiLoad 26/600 Superdex 75 pg column equilibrated with 20 mm Tris, 150 mm KCl, 5% glycerol, and 1 mm TCEP, pH 7.5. Purification of proteins were followed at every step using SDS-PAGE.

An Octet RED96 system and streptavidin (SA)-coated biosensors (FortéBio) were used to measure association and dissociation kinetics for HMT-Gpsm2 in the presence and absence of Gα_t_*. Binding studies were performed in 20 mm Tris, 150 mm KCl, 5% glycerol, 1 mm TCEP, 0.5 mg/ml BSA, pH 8.0 with and without 12 μm Gα_t_*. All steps were performed at 26°C, with biosensors stirred into 0.2 ml of sample in each well at 1000 rpm, and at a data acquisition rate of 5.0 Hz. His-Avi-Trx-Frmpd1-pept (biotinylated in *E. coli* by native BirA ligase) was loaded onto SA sensors at a concentration of 0.02 mg/ml for 120 s. Data for association and dissociation phases of the assay were collected as shown in Results section. To correct for baseline drift and nonspecific binding, reference sensors lacking bound His-Avi-Trx-Frmpd1-pept were used in the BLI assays with HMT-Gpsm2 protein at the highest concentrations. Kinetic data fitting was performed using FortéBio Data Analysis software 10.0. Steady-state data fitting was performed using GraphPad Prism 7 software with the equation for one site-specific binding.

### *In vitro* Gα_t_* pull down experiments

The C-terminal FLAG tag was introduced into the HMT-Gpsm2 using site directed mutagenesis. The Avi tag of the His-Avi-Trx-Frmpd1-pept was replaced with an HA tag. The Avi-tagged Gα_t_* construct was described previously (Srivastava et al., 2020). Proteins were expressed and purified similarly as described above. MBP tag was removed from Gpsm2 after an Ni affinity purification step using TEV protease, the sample was dialyzed in 20 mm Tris buffer (pH 7.5) containing 5% glycerol, 25 mm NaCl, 5 mm β-mercaptoethanol. Gpsm2 was purified by passing through HiTrapQ anion exchange chromatography followed by purification by SEC using HiLoad 16/600 Superdex 200 pg column equilibrated with 20 mm Tris, 150 mm KCl, 5% glycerol, 1 mm TCEP, pH 7.5. Avi-tagged Gαt* was purified by SEC after Ni affinity purification. For pull down experiments, Avi tagged Gα_t_* was bound to streptactin-agarose beads (ibi) by incubating in 20 mm Tris, 150 mm KCl, 5% glycerol, 1 mm TCEP, 0.5 mg/ml BSA, pH 7.5 (buffer P) for 30 min at 4°C. Beads were washed three times in buffer P and incubated with 10 μm Gpsm2-FLAG and/or 20 μm of His-HA-thioredoxin-Frmpd1 peptide. As a control, streptactin beads in absence of Avi-Gα_t_* were incubated with 10 μm Gpsm2-FLAG and/or 20 μm of His-HA-thioredoxin-Frmpd1 peptide. To rule out the interaction between Frmpd1 peptide and Avi tagged- Gα_t_*, pull down experiment was also performed using the Avi-tagged- Gα_t_* and 20 μm of His-HA-thioredoxin-Frmpd1 peptide.

## Results

### *Frmpd1* localizes to rod inner segments and synapses

We have previously demonstrated that *Frmpd1* RNA transcripts in rod photoreceptor cells are generated from an alternate promoter and that the deletion of rod-specific untranslated *Frmpd1* exon 1a in mice (*Frmpd1^Δ1a^*) resulted in complete loss of Frmpd1 expression only in rods (Campla et al., 2019). Immunohistochemical staining of *Frmpd1^Δ1a^* mouse retina using multiple cell type-specific protein markers did not reveal any gross defect in retinal lamination or synaptic morphology in outer plexiform layer (OPL; Figs. 1, 2).

**Figure 3.**
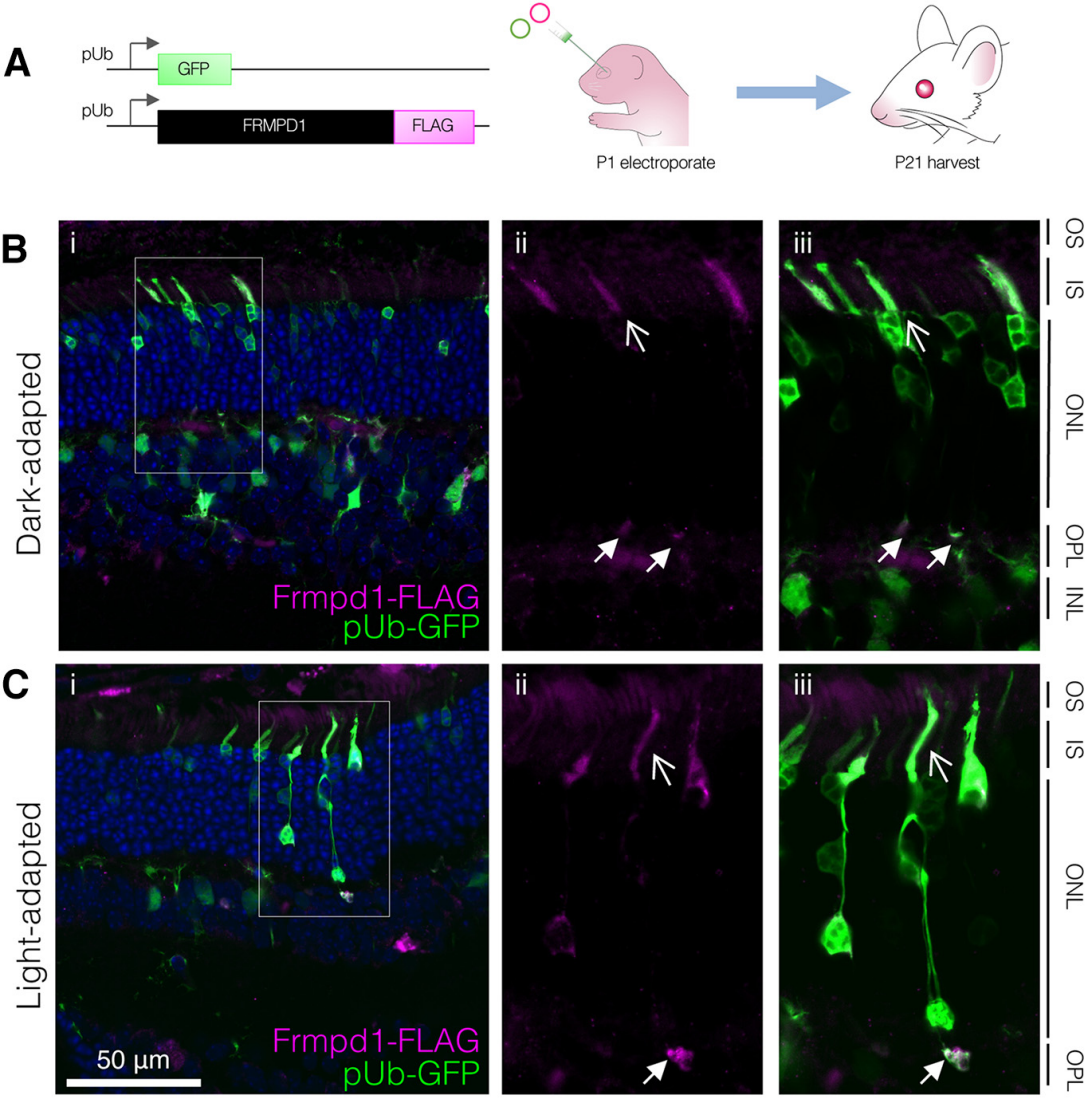
Frmpd1 localizes to rod inner segments and synapses. ***A***, *In vivo* electroporation workflow. FLAG-tagged full-length Frmpd1 and GFP expression constructs under control of ubiquitin promoter (pUb) were coinjected subretinally in neonatal mouse pups at P1 and introduced to the retina via electroporation. At P21, eyes were harvested and processed for immunohistochemistry. ***B***, Frmpd1 localization in dark-adapted retina. Mice were dark-adapted overnight before eyes were harvested and processed for immunohistochemistry to detect both GFP and FLAG-tagged Frmpd1 protein using anti-FLAG antibody (i). Immunofluorescence staining for the FLAG-Frmpd1 shows clear localization to both the rod inner segments (open arrowheads) and synapses (closed arrowheads; ii, iii). ***C***, Frmpd1 localization in light-adapted retina. Mice were light-adapted for 1 h before eyes were harvested and processed for immunohistochemistry to detect both GFP and FLAG-tagged Frmpd1 protein using anti-FLAG antibody (i). As in dark-adapted retina, FLAG-Frmpd1 immunofluorescence staining localized to both rod inner segments (open arrowheads) and synapses (closed arrowheads; ii, iii). OS, outer segment; IS, inner segment; ONL, outer nuclear layer; OPL, outer plexiform layer; INL, inner nuclear layer. Scale bar: 50 μm.

Commercial and custom-generated antibodies against Frmpd1 showed a diffuse nonspecific immunohistochemical staining in both wild-type (WT) and *Frmpd1^Δ1a^* retina, making them unsuitable for localization studies. For this reason, a FLAG-tagged Frmpd1 expression construct was instead introduced into neonatal CD1 retinal progenitor cells by *in vivo* electroporation to determine Frmpd1 protein localization at postnatal day (P)21 (Fig. 3*A*). Previous studies have shown that *in vivo* electroporation of neonatal rodent retina with pUb-GFP results in a GFP-positive cell population that is ∼80% rod photoreceptors and ∼15% bipolar cells (Matsuda and Cepko, 2004), which roughly mirrors the expression profile of endogenous *Frmpd1* in the retina (Campla et al., 2019). Since some retinal proteins are known to exhibit light-dependent redistribution (Arshavsky, 2003; Calvert et al., 2006), we evaluated whether Frmpd1-FLAG localization differs based on light/dark-adapted state of the retina. Electroporated mice were thus either dark-adapted overnight (Fig. 3*B*) or exposed to bright light for 1 h (Fig. 3*C*) before processing for immunohistochemistry. Regardless of the light adaptation state, Frmpd1-FLAG was consistently detected in the photoreceptor inner segment region and outer plexiform layer (OPL) of the retina. Although exogenous expression of proteins has the potential to disrupt natural cell morphology, the well-circumscribed Frmpd1-FLAG staining in the OPL is most consistent with rod spherule presynaptic morphology as compared with dendritic processes of other cell types that also make projections into the OPL. The inner segment staining also likely represents that of rods since cones are not typically transfected during *in vivo* electroporation. Together, these results suggest that Frmpd1 localizes to the inner segments and synapses of rod photoreceptors, which is consistent with the proposed scaffolding function of Frmpd family proteins (Moleirinho et al., 2013).

### Frmpd1 forms complexes with Gpsm2 in the retina

**Figure 4.**
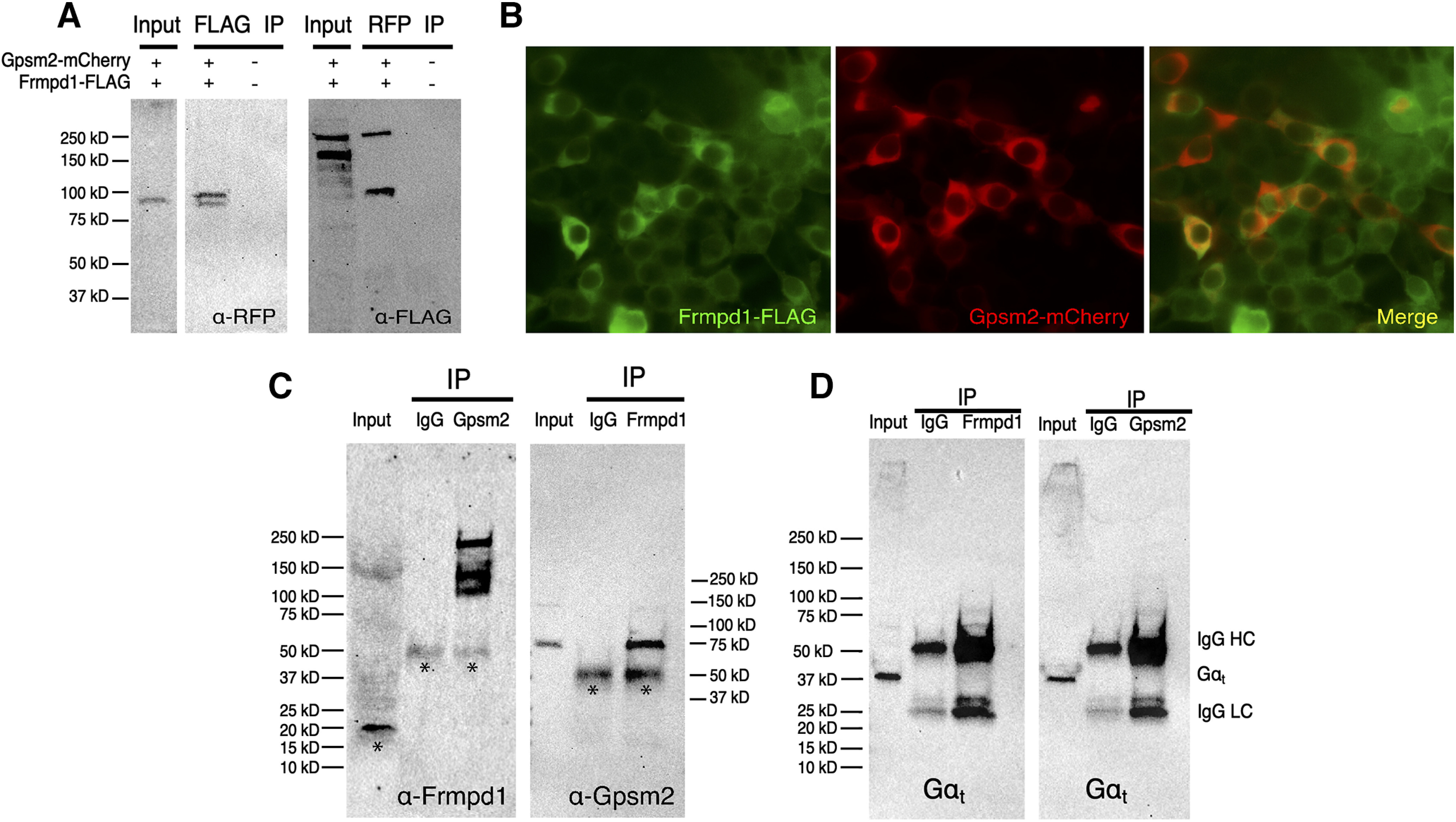
Frmpd1 interacts with Gpsm2 in the retina. ***A***, Co-Immunoprecipitation (Co-IP) of Frmpd1 and Gpsm2. HEK293 cells were co-transfected with 3′ FLAG-Frmpd1 and mCherry-Gpsm2 expression constructs. Frmpd1-containing protein complexes were immunoprecipitated with anti-FLAG antibody and probed with anti-RFP (mCherry) to detect Gpsm2. Gpsm2-containing protein complexes were immunoprecipitated with anti-RFP antibody and probed with anti-FLAG to detect Gpsm2. ***B***, Colocalization of Frmpd1 and Gpsm2 in HEK293 cells. HEK293 cells were co-transfected with 3′ FLAG-Frmpd1 and mCherry-Gpsm2 expression constructs and stained with anti-FLAG (green) antibody to visualize Frmpd1. Gpsm2 is visualized by mCherry protein fluorescence (red). ***C***, Left panel, IP of Gpsm2 complexes from retina. Mice were light-adapted for 1 h, then dark-adapted for 1 h. Gpsm2-containing protein complexes were immunoprecipitated from retina lysate using anti-Gpsm2 antibody; 10% of input lysate and 50% of immunoprecipitated protein complexes were processed for immunoblotting with anti-Frmpd1 antibody. Asterisks (*) represent nonspecific band staining. Right panel, IP of Frmpd1 complexes from retina. Lysates were prepared as described, and Frmpd1-containing protein complexes were immunoprecipitated from retina lysate using anti-Frmpd1 antibody; 10% of input lysate and 50% of immunoprecipitated protein complexes were processed for immunoblotting with anti-Gpsm2 antibody. Asterisks (*) represent nonspecific band staining. ***D***, Immunoblot of proteins pulled down from retina lysates with anti-Frmpd1 (left panel) and anti-Gpsm2 (right panel) antibodies and probed with Gαt antibodies.

**Figure 5.**
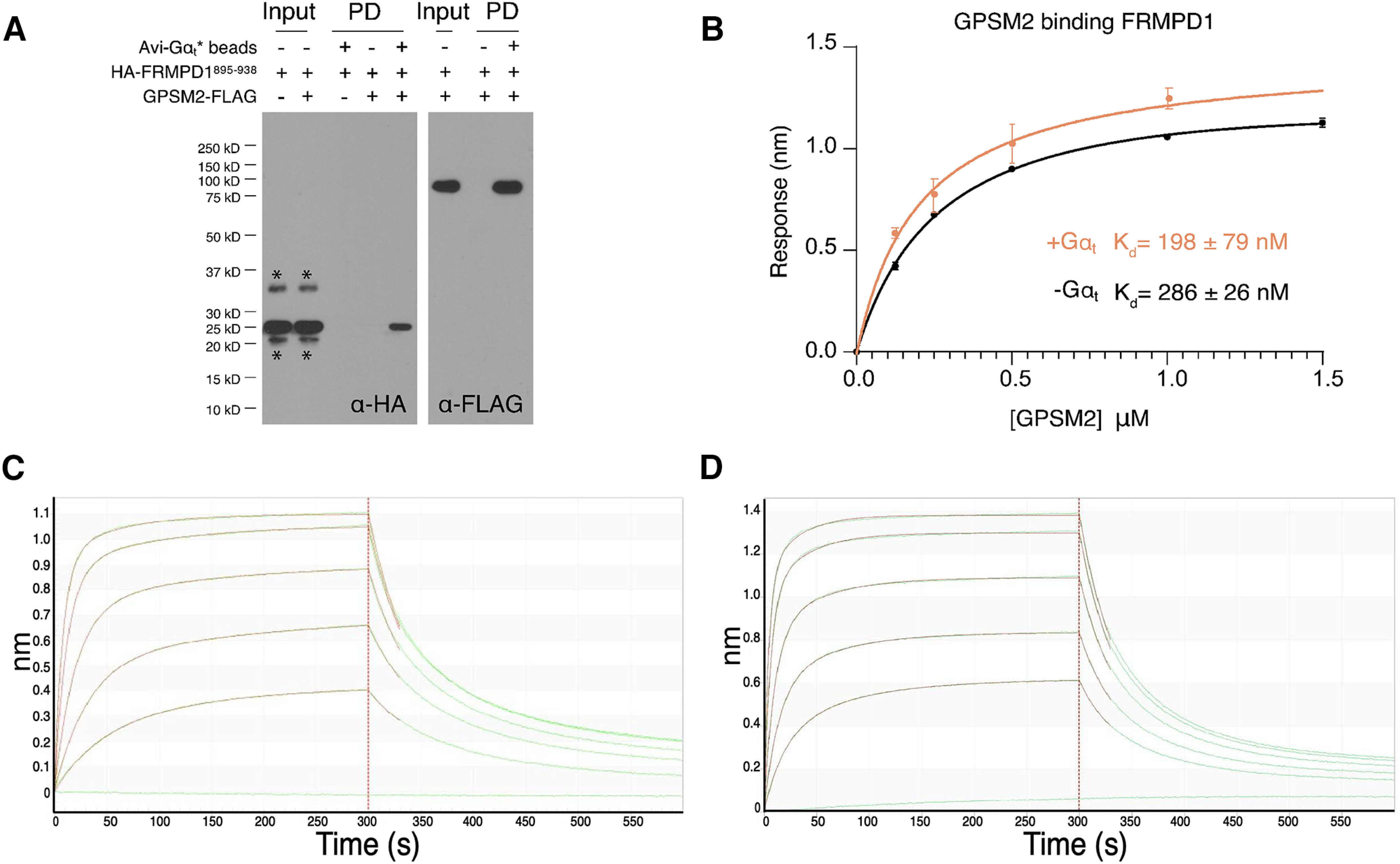
Frmpd1 forms a ternary complex with Gpsm2 and Gαt. ***A***, *In vitro* interaction of Frmpd1 with Gpsm2 and Gα_t_*. Streptavidin resin beads with (+) and without (–) bound Avi-Gαt* were incubated with Gpsm2-FLAG and/or HA-thioredoxin tagged Frmpd1 fragment (residues 895–938 previously shown to contain Gpsm2 binding site). Immunoblots of pull down (PD) products from beads with (+) and without (–) bound Avi-Gαt* in presence of HA-thioredoxin-tagged Frmpd1 and/or Gpsm2-FLAG were probed with anti-HA and anti-FLAG antibodies. ***B***, Steady state analysis of the Bio-Layer Interferometry (BLI) data (as shown in Fig. 5) for the binding of Gpsm2 to the Avi-Trx tagged Frmpd1 895–938 coupled to a streptavidin biosensor in the presence (+) or absence (–) of Gα_t_*. Error bars show SEM. ***C***, Kinetics of association and dissociation for Gpsm2 and Avi-thioredoxin tagged Frmpd1 895–938 coupled to a streptavidin biosensor as determined using Bio-Layer Interferometry (BLI). Representative curves are shown for black fitting in ***A***. ***D***, Kinetics of association and dissociation for Gpsm2 and Avi-thioredoxin-tagged Frmpd1 895–938 coupled to a streptavidin biosensor in the presence of Gαt* as determined using BLI. Representative curves are shown for orange fitting in ***A***.

Gpsm2 acts as a guanine nucleotide dissociation inhibitor (GDI) that preferentially interacts with and stabilizes the inactive, GDP-bound form of G-protein α subunits, including Gα_t_ (Kerov et al., 2005a; Nair et al., 2005a). An interaction between Gpsm2 and Frmpd1 has been well-characterized *in vitro* (Pan et al., 2013). Indeed, IP from HEK293 cells cotransfected with 3′ FLAG-Frmpd1 and mCherry-Gpsm2 further validate this interaction (Fig. 4*A*), supported by the colocalization of both proteins in transfected cells (Fig. 4*B*). Gpsm2 is expressed in rod photoreceptors and shown to localize to rod IS and synapses where it has the potential to interact with Frmpd1 (Kerov et al., 2005b; Nair et al., 2005a). Since the function of Frmpd1 and its interaction with Gpsm2 *in vivo* is unknown, we next performed IP of Gpsm2-containing protein complexes from retina lysates. In accordance with our cell culture results, Frmpd1 could be identified in the Gpsm2-bound fraction (Fig. 4*C*). Additional bands detected by anti-Frmpd1 antibody may represent nonspecific staining, degradation products, or multiple isoforms of Frmpd1 protein (Fig. 4*C*). A reciprocal IP of Frmpd1-containing protein complexes from retinal lysate yielded Gpsm2 in the Frmpd1-bound fraction, thereby confirming an Frmpd1-Gpsm2 interaction in the retina (Fig. 4*C*). Gα_t_ was not detected in Gpsm2 or Frmpd1-bound fractions (Fig. 4*D*). Since the interaction between Gpsm2 and Gαt in the retina has been previously documented (Kerov et al., 2005a, b; Nair et al., 2005a, b), it is possible that in our assay the Gpsm2 antibody blocked Gαt-binding site on Gpsm2. We therefore performed an *in vitro* pull down assay using purified proteins to further characterize interactions between Frmpd1, Gpsm2, and Gα_t_ (Fig. 5*A*). Avi-tagged Gαt* was immobilized on streptavidin resin and incubated with FLAG-tagged Gpsm2 and HA-tagged Frmpd1 peptide (residues 895–938, previously shown as Frmpd1-Gpsm2 binding site; Pan et al., 2013). Importantly, HA- and thioredoxin-tagged Frmpd1 fragment was pulled down by immobilized avi-tagged Gα_t_ only in the presence of Gpsm2-FLAG (lane 5 of 5A α-HA, lane three of 5A α-FLAG). Thus, we confirmed the interaction between Gpsm2, and Gα_t_ and show a ternary complex of Gpsm2 with Frmpd1 and Gα_t_.

Cell culture studies have demonstrated that the interaction between Frmpd1 and Gpsm2-related protein Gpsm1 (G-protein signaling modulator 1, also called AGS3) is lost in the presence of Gα_t_, with Gpsm1-Frmpd1 and Gpsm1-Gα forming mutually exclusive complexes (An et al., 2008). To determine whether the interaction between Frmpd1 and Gpsm2 is similarly influenced by Gα_t_, Frmpd1-Gpsm2 binding assays were performed in the absence and presence of Gα_t_ (Fig. 5*B–D*). Interestingly, Gpsm2-Frmpd1 steady state binding did not differ significantly between the two conditions, suggesting that in contrast to Gpsm1, Frmpd1-Gpsm2 binding is not dependent on Gα_t_ concentration. Altogether, the data in Figure 5 demonstrate the formation of the ternary complex, whereby Frmpd1 and Gαt noncompetitively bind to the TPR-domain and GoLoco repeats of Gpsm2, respectively. This ternary complex parallels the Numa-Gpsm2-Gαi complex in asymmetric cell division, where Numa interacts with the N-terminal TPR domain of Gpsm2, while Gαi interacts with the Gpsm2 C terminal GoLoco motifs of Gpsm2 (Du and Macara, 2004).

### Rod photoreceptors in *Frmpd1^Δ1a^* and Gpsm2^−/−^ mice display normal light sensitivities

**Figure 6.**
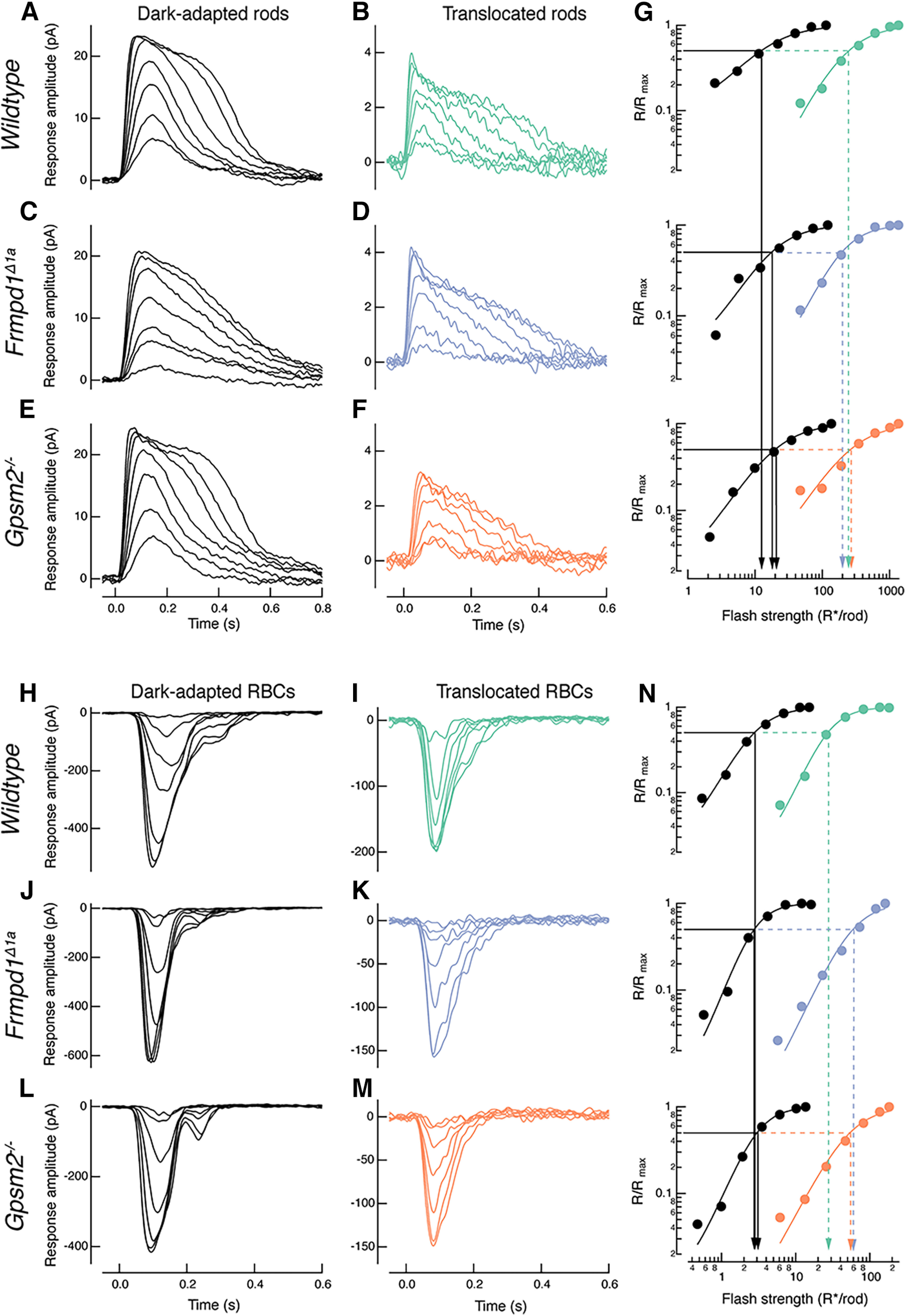
Rod bipolar cells (RBCs) light responses in *Frmpd1^Δ1a^* and Gpsm2^−/−^ mice display decreased sensitivity compared with wild-type during Gα_t_ translocation. ***A***, ***C***, ***E***, Representative dark-adapted response families of wild-type (***A***), *Frmpd1^Δ1a^* (***C***), and Gpsm2^−/−^ (***E***) rods. ***B***, ***D***, ***F***, Representative light response families of wild-type (***B***), *Frmpd1^Δ1a^* (***D***), and Gpsm2^−/−^ (***E***) rods, following the Gα_t_ translocation protocol. ***G***, Intensity-response relationships for dark adapted (DA) (black) and translocated (wild-type, green; *Frmpd1^Δ1a^*, blue; Gpsm2^−/−^, orange) rods. Solid and dashed arrows indicate half maximal flash strength (I_1/2_) before and after translocation, respectively. ***H***, ***J***, ***L***, Representative dark-adapted response families of wild-type (***H***), *Frmpd1^Δ1a^* (***J***), and Gpsm2^−/−^ (***L***) RBCs. ***I***, ***K***, ***M***, Representative response families of wild-type (***I***), *Frmpd1^Δ1a^* (***K***), and Gpsm2^−/−^ (***M***) RBCs following the Gα_t_ translocation protocol. ***N***, Intensity-response relationships for DA and translocated RBCs with color coding as in ***G***. Solid and dashed arrows indicate half maximal flash strength (I_1/2_) before and after translocation, respectively.

To investigate physiological roles of Frmpd1 and Gpsm2 in the retina, we performed functional evaluation of rod photoreceptors by recording light-evoked responses of wild-type, *Frmpd1^Δ1a^* and Gpsm2^−/−^ rods from dark-adapted retinal slices (Fig. 6*A*,*C*,*E*). Flash families from single rods were recorded using the whole-cell patch-clamp technique in voltage clamp mode. Responses evoked by increasing light intensities displayed robust hyperpolarizing outward currents in both transgenic genotypes with no major differences in response properties when compared with wild-type rods, indicating normal phototransduction in all three genotypes (Fig. 6*A*,*C*,*E*,*G*; Table 1). The sensitivity, amplitude and time to peak of the rod responses between the genotypes were similar (see Table 1). To examine functional effects of the interaction between Frmpd1-Gpsm2 when Gα_t_ localizes to the inner segment and synapse where it has the potential to transiently interact with these proteins, retinas were exposed to a light induced Gα_t_ translocation (TS) protocol as previously described (Majumder et al., 2013). As predicted, following the TS protocol, rods of each genotype showed a major >10-fold desensitization with faster response kinetics and reduced amplitude, characteristic of light adaptation. The sensitivities of both *Frmpd1^Δ1a^* (177.6 ± 7.8 R*/rod, *N* = 4; *p* = 0.62) and Gpsm2^−/−^ rods (206 ± 14.6 R*/rod, *N* = 3; *p* = 0.38) were similar compared with wild-type rods (200.6 ± 11 R*/rod, *N* = 5; Fig. 6*B*,*D*,*F*,*G*; Table 1), demonstrating no major functional differences in the response properties of translocated rods in the absence of Frmpd1 or Gpsm2 in fully dark-adapted or bright light conditions (Fig. 6*G*; Table 1).

### Lack of Frmpd1 or Gpsm2 alters rod bipolar cell sensitivity in bright light conditions

Under bright light conditions, activated Gα_t_^GTP^ translocates from rod outer segments and redistributes to inner segments and synapses, where it can further modulate synaptic activity (Majumder et al., 2013). As indicated by immunohistochemistry and binding assay data, Frmpd1 has the potential to colocalize and interact with Gpsm2 and Gpsm2-Gα_t_ in rod IS and synapses following Gα_t_ translocation (see Figs. 3-5). We therefore investigated the effect of Frmpd1 and Gpsm2 on synaptic transmission. To this end, we examined the transmission of light signals from rods to rod bipolar cells (RBCs), the first order neuron that conveys the rod response to the rest of the retina. We initially assessed functionality of wild-type, *Frmpd1^Δ1a^* and Gpsm2^−/−^ RBCs by comparing their light responses in dark-adapted conditions (Fig. 6*H*,*J*,*L*). Dark-adapted responses in both *Frmpd1^Δ1a^* and Gpsm2^−/−^ RBCs displayed robust depolarizing inward currents, characteristic of healthy RBCs (Fig. 6*H*,*J*,*L*). The response properties of RBCs between genotypes did not show any major differences in sensitivity, the amplitude and the time to peak in the absence of either Frmpd1 or Gpsm2, thus displaying normal function and synaptic transmission in dark-adapted conditions (Fig. 6*N*; Table 1). However, there was a significant >twofold reduction in the sensitivity of RBCs in both *Frmpd1^Δ1a^* (57.3 ± 3.3 R*/rod, *N* = 4; *p* = 0.0001) and Gpsm2^−/−^ (48.7 ± 5.4 R*/rod, *N* = 5; *p* = 0.0016) compared with wild-type (19 ± 3.4 R*/rod, *N* = 5) following the Gα_t_ translocation (TS) protocol. This decrease in RBC sensitivity in the absence of either Frmpd1 or Gpsm2 in the rod synapse suggests that signal transmission from photoreceptors to RBCs is affected in bright light conditions, when Gα_t_ is also localized to the rod IS and synapse. Our data thus indicate that the interaction of Frmpd1 and Gpsm2 in association with Gα_t_ in the normal wild-type retina is required for enhancing synaptic transmission from rods to RBCs on light adaptation.

### Frmpd1 expedites the trafficking of Gα_t_ from rod inner segments and synapses

**Figure 7.**
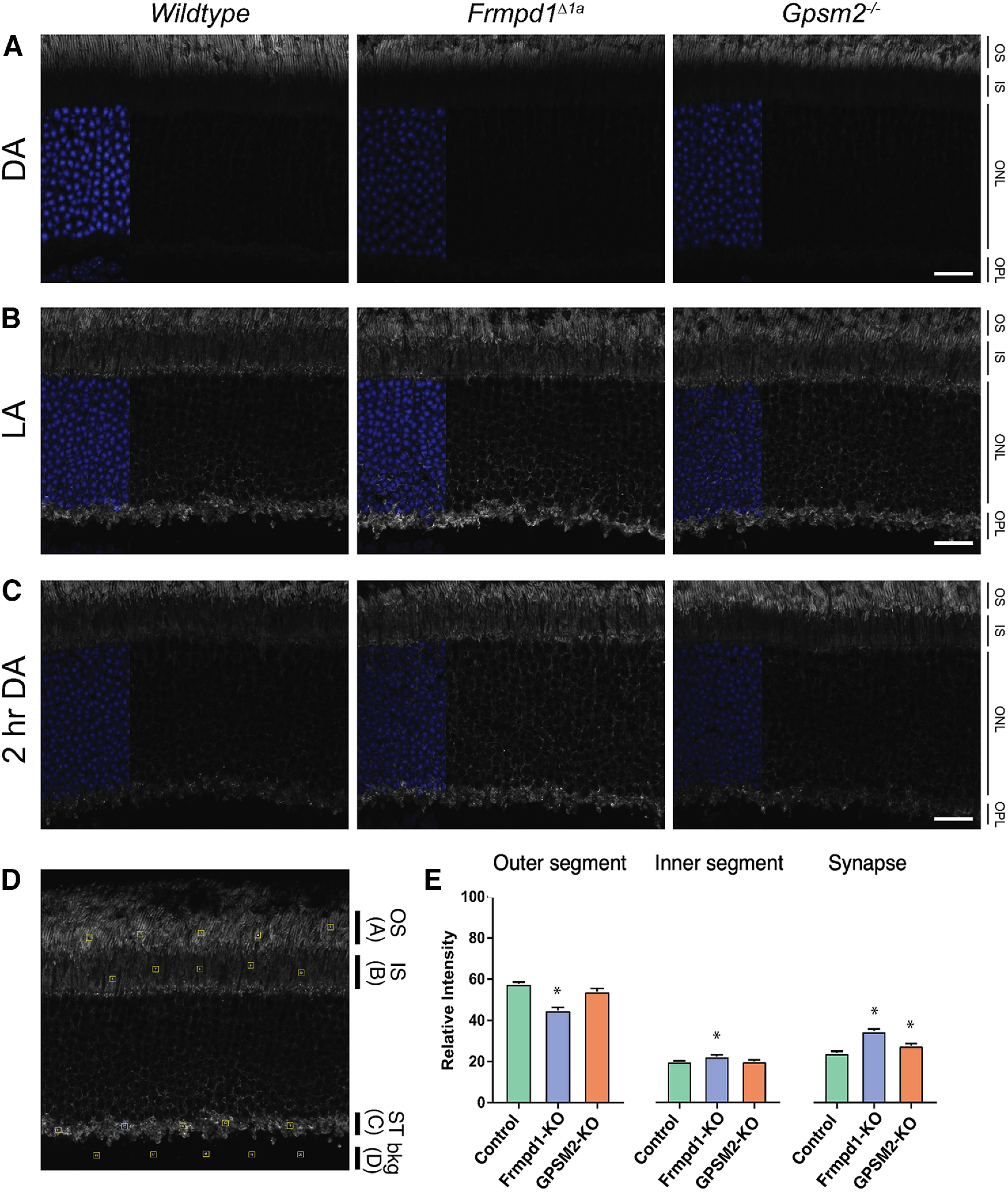
Frmpd1 facilitates dark-adapted (DA) return of Gα_t_ to rod outer segments. ***A***, Wild-type, *Frmpd1^Δ1a^*, and *Gpsm2*−/− mice were DA overnight, after which eyes were harvested and processed for immunohistochemistry to detect Gα_t_ in DA retina. ***B***, DA mice were exposed to bright light (∼1000 lux) for 1.5 h to detect Gα_t_ translocation in light-adapted (LA) retina. ***C***, Light-adapted mice were placed in darkness for 2 h to detect Gα_t_ in the retina during the course of dark adaptation (2-h DA). ***D***, Example of transducin quantification for immunofluorescence preparations. Five equal-sized squares (regions of interest, ROIs) (yellow square) for outer segment (OS; A) (***A***), inner segment (IS; B) (***B***), synaptic terminal (ST; C) and background (bkg; D) were chosen for each image analyzed (see Materials and Methods, Transducin quantification assay). The total fluorescence (*F*_tot_) was estimated by summing the fluorescence (F) values of the ROIs within the three relevant photoreceptor layers *F*_tot_ = (A – D) + (B – D) + (C – D). Relative intensity of each layer was then calculated as a ratio of the total: outer segments fluorescence (*F*_OS_) = (A − D)/*F*_tot_; inner segments fluorescence (*F*_IS_) = (B − D)/*F*_tot_; synaptic terminal fluorescence (*F*_ST_) = (C − D)/*F*_tot_. ***E***, Quantification of relative intensity of the retinal layers at 2-h DA. Statistical significance is denoted with an asterisk where *p* < 0.05. OS, outer segment; IS, inner segment; ONL, outer nuclear layer; OPL, outer plexiform layer. Scale bar: 20 μm.

Currently, only a few interacting partners of Gα_t_ have been identified outside of the phototransduction cascade, and some of these have functions related to the movement of Gα_t_ between IS/OS (Gopalakrishna et al., 2011; Zhang et al., 2011; Sinha et al., 2013; Chaya et al., 2019; Srivastava et al., 2020). Since Gα_t_ translocates to the rod IS and synapse on bright light exposure where Frmpd1 and Gpsm2 are positioned, we wondered whether Frmpd1 and/or Gpsm2 could influence Gα_t_ translocation dynamics between these subcellular compartments during dark adaptation *in vivo*. To test this, Gpsm2^−/−^ and *Frmpd1^Δ1a^* mice were first light-adapted to cause translocation of Gα_t_ from the OS to the IS and synapses (Fig. 7*B*), followed by 2 h of dark adaptation to allow for the return of Gα_t_ to OS (Fig. 7*C*). Interestingly, the return of Gα_t_ was markedly delayed in *Frmpd1^Δ1a^* (and to a slight degree in Gpsm2^−/−^) rods after 2 h of dark adaptation, as evidenced by the persistence of Gα_t_ staining in synaptic terminals (Fig. 7*C*,*E*). These results suggest a necessary role for Frmpd1 in mediating the timely return of Gα_t_ to rod OS following light-induced translocation.

### Lack of Frmpd1 or Gpsm2 alters response recovery after bright light exposure

**Figure 8.**
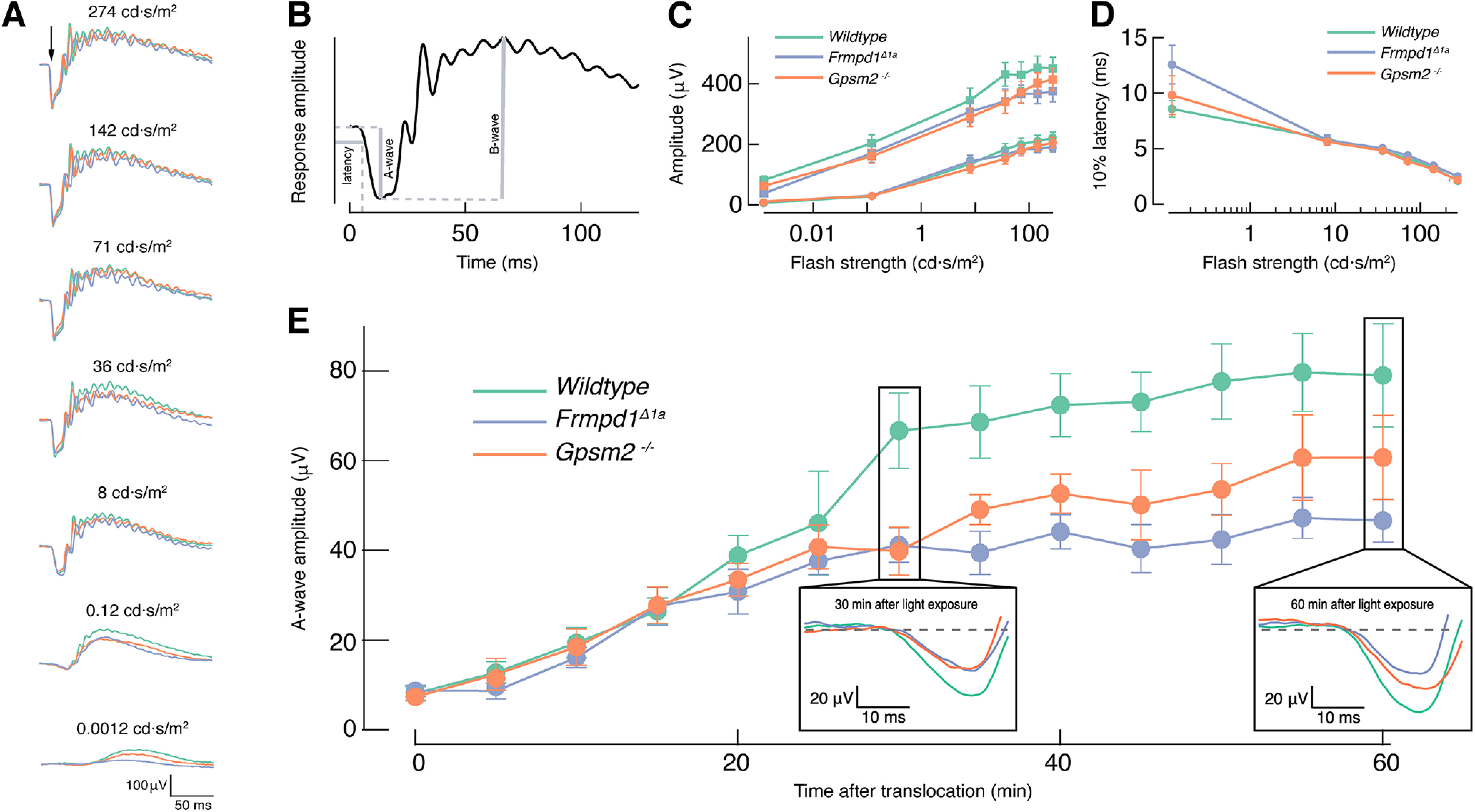
Response recovery and dark adaptation is compromised in *Frmpd1^Δ1a^* and *Gpsm2*−/− mice. ***A***, Mean dark-adapted electroretinogram (DA ERG) responses to 20-ms light flashes (arrow) of increasing strength for wild-type (green), *Frmpd1^Δ1a^* (blue), and *Gpsm2*−/− (orange) mice. ***B***, Schematics of ERG parameters extraction: the latency (as time for the response to reach 10% of the maximum value), a-wave amplitude (from response baseline to the lowest deflection point), and b-wave amplitude (from the lowest deflection point to the maximum value). ***C***, Intensity-response relationship of the dark-adapted (DA) a-wave (circles) and b-wave (squares) amplitudes. ***D***, 10% latency of the DA a-wave as a function of light intensity. ***E***, Recovery of the scotopic *in vivo* ERG a-wave response amplitude following the Gα_t_ translocation protocol. Insets shows representative *in vivo* ERG a-wave responses at 30 and 60 min.

To examine functional consequences of the delayed return of Gα_t_ to the OS in *Frmpd1^Δ1a^* rods during dark adaptation, we next performed *in vivo* ERG recordings to study the physiology of dark adaptation of the retina as a whole (Fig. 8). As a first assessment of the physiological state of the retina *in vivo*, we recorded scotopic photoresponses from dark-adapted wild-type, *Frmpd1^Δ1a^*, and Gpsm2^−/−^ mice. The ERG a-wave (predominantly rod-dominated) and b-wave (downstream circuitry) across all three genotypes displayed similar response properties, confirming normal function of transgenic retinas in the dark-adapted state (Fig. 8*A–D*). Following light-induced translocation of Gα_t_, the initial dark adaptation and recovery of a-wave amplitude were similar in all genotypes, presumably because of visual pigment regeneration. However, after ∼30 min, there was a significant reduction in the a-wave amplitude recovery in both *Frmpd1^Δ1a^* (40 ± 4 μV, *N* = 7; *p* = 0.006) and Gpsm2^−/−^ (44.5 ± 3.4 μV, *N* = 9; *p* = 0.012) compared with wild-type (70.8 ± 8, *N* = 9) animals (Fig. 8*E*). At 55–60 min time point, the a-wave was significantly smaller only for Frmpd1*^Δ1a^* mice (46.2 ± 4.1 μV, *N* = 7; *p* = 0.0007) compared with wild type (86.5 ± 7.7, *N* = 8). The significant lack of relative a-wave recovery (Fig. 8*E*) in *Frmpd1^Δ1a^* mice after 60 min is consistent with the observed delayed return of Gα_t_ to the OS of *Frmpd1^Δ1a^* rods following bright light exposure (see Fig. 7), indicating a functional role for Frmpd1 accelerating retrograde transport of Gα_t_ back to OS during dark adaptation.

## Discussion

Redistribution of key phototransduction components within photoreceptor compartments is critical for adaptation, survival, and effective synaptic signaling in response to varying light intensities. However, despite extensive investigations, the precise mechanism of light induced translocation remains poorly understood and controversial (Nair et al., 2005b; Peterson et al., 2005; Karan et al., 2008; Reidel et al., 2008; Kerov and Artemyev, 2011; Zhang et al., 2011; Majumder et al., 2013; Frederick et al., 2020; Srivastava et al., 2020). Here, we provide novel insights into the mechanisms of Gα_t_ trafficking and synaptic transmission in rod photoreceptors. We show that Frmpd1 is required for the efficient trafficking of Gα_t_ back to the rod OS in dark conditions following light-induced translocation, and propose a mechanism of protein-facilitated transport that may act in concert with passive diffusion. We further demonstrate that the absence of Frmpd1 and/or Gpsm2 in rods results in impaired rod to RBC synaptic transmission in bright light conditions, suggesting their importance in optimizing rod signaling. Our results provide support for coordination of Gα_t_ trafficking and synaptic signaling, which is facilitated by interaction of the scaffolding protein Frmpd1 with G-protein modulator Gpsm2.

Light-induced translocation of Gα_t_ to the rod presynaptic terminal (spherule) has previously been shown to enhance rod to RBC synaptic transmission, yet the mechanism through which this is accomplished remains unknown (Majumder et al., 2013). In the absence of Frmpd1 or Gpsm2, a greater light stimulus is required to evoke comparable responses from RBCs compared with the wild-type in the Gα_t_ translocated state, despite no detectable differences in the dark-adapted state. Our data suggest that Frmpd1 and Gpsm2 are each required for optimizing rod to RBC transmission when Gα_t_ is present in the rod spherule. We note that *Frmpd1^Δ1a^* mice have a specific loss of *Frmpd1* in rod photoreceptors but expression of *Frmpd1* is still detectable in RBCs (Campla et al., 2019), providing clear evidence of this optimization effect being mediated presynaptically. The involvement of Frmpd1 and Gpsm2 in the transport of Gα_t_ back to the OS is evidenced by the delayed return of Gα_t_ to OS in *Frmpd1^Δ1a^* mice and delayed functional recovery of the rod-mediated photoresponse in *Frmpd1^Δ1a^* and *Gpsm2*−/− mice during dark adaptation. The localization of Frmpd1 and Gpsm2 to rod IS and synaptic terminals optimally positions these molecules for influxes of activated Gα_t_ on bright light exposure. Gα_t_ needs to be GDP-bound to form a heterotrimer with Gβγ_t_ en route to the OS and becomes associated with the disk membrane because of dual lipidation of the trimeric G protein (Frederick et al., 2020). This may be accelerated in part by Gpsm2, which preferentially loads and stabilizes multiple Gα_t_^GDP^ molecules using GoLoco motifs (McCudden et al., 2005). It is possible that Frmpd1, via its membrane scaffolding function, could also augment this process, thereby facilitating a faster recovery after light exposure.

We propose that Gpsm2 scavenges and sequesters newly inactivated Gα_t_^GDP^ from the cytosol in inner segments and synapses and interacts with Frmpd1 near the plasma membrane to facilitate Gα_t_^GDP^ trafficking back to OS. Gpsm2 is known to interact with dynein/dynactin to activate motor activity pulling on astral microtubules during mitosis (Zheng et al., 2013; Pirovano et al., 2019). Moreover, a postmitotic role for Gpsm2 has been described in auditory and vestibular hair cells, where it localizes to the tips of stereocilia to modulate neuronal outgrowth (Mauriac et al., 2017). Given that membrane association and scaffolding functions have been described for Frmpd family proteins (Moleirinho et al., 2013), Frmpd1 likely plays a role in anchoring Gpsm2-Gα_t_^GDP^ to plasma membranes of IS and synapses, further expediting the Gα_t_^GDP^ sorting through the connecting cilium. We hypothesize that Frmpd1-Gpsm2-mediated trafficking of Gα_t_ toward the connecting cilium may be accomplished through retrograde transport along microtubule filaments. This is supported in part by the suggested role of the cytoskeleton in light-dependent trafficking of proteins, with inhibition of microtubules specifically slowing the return of Gα_t_ during dark adaptation (Peterson et al., 2005; Reidel et al., 2008). Although some Gα_t_ may return to the OS by UNC119-mediated diffusion and/or Arl3-mediated sorting (Hanke-Gogokhia et al., 2016; Frederick et al., 2020), our proposed Frmpd1-Gpsm2 mediated trafficking provides an additional transport mechanism to further accelerate this process for proper restoration of the rod photoresponse. We note that Gα_t_ trafficking back to OS during dark adaptation was more strikingly affected in the absence of Frmpd1. It is possible that Gpsm1 could compensate for the loss of Gpsm2 function in rods since Gpsm1 is also expressed in these cells (Kim et al., 2016; Zelinger et al., 2017) and can interact with Frmpd1 (An et al., 2008). Unlike Gpsm2, Gpsm1 is not expressed in RBCs (Dhingra et al., 2008). Thus, there could be compensatory mechanisms taking place at the level of the synapse, involving Frmpd2 and Gpsm1 isoforms that would allow transducin to return to the OS, but with a slower time course. Moreover, there could be other proteins involved in synaptic release but not in the return of transducin to the OS of the rods. The changes in the RBCs sensitivity between the WT and the knock-outs (KOs) could be a consequence of altered protein expression in the synapse of the two KOs, which could affect the mechanism of vesicle release. Interactors of Gpsm2 and Frmpd1 in rod bipolar cells would provide an interesting avenue for future investigation, as there are likely cell type-specific functions apart from those reported here in rod photoreceptors.

In summary, our studies demonstrate Frmpd1 as a key modulator of rod synaptic signaling and retrograde transport of Gα_t_ during dark adaptation through its interaction with Gpsm2 at the rod presynapse. We propose that this protein-mediated retrograde transport mechanism works alongside passive diffusion to further accelerate the return of Gα_t_ en masse to the OS. Identification of potential interactors of Frmpd1 (and of Gpsm2 and Gpsm1) that likely constitute multi-protein complexes at the rod synaptic terminal provides an attractive area of future investigations to elucidate how Gα_t_ exerts its effects on synaptic transmission to rod bipolar cells.

## Synthesis

Reviewing Editor: Matthew Grubb, King’s College London Decisions are customarily a result of the Reviewing Editor and the peer reviewers coming together and discussing their recommendations until a consensus is reached. When revisions are invited, a fact-based synthesis statement explaining their decision and outlining what is needed to prepare a revision will be listed below. The following reviewer(s) agreed to reveal their identity: Jeannie Chen, Peter Calvert. Note: If this manuscript was transferred from JNeurosci and a decision was made to accept the manuscript without peer review, a brief statement to this effect will instead be what is listed below.

Reviewer 1:

The authors have adequately addressed previous concerns.

Reviewer 2:

"Frmpd1 facilitates trafficking of G-protein transducin and modulates synaptic function in rod photoreceptors of mammalian retina", by Campla et al. is a revised resubmission. The authors provide evidence that Frmpd1 and Gpsm2 form a trimeric complex with the G protein, transducin, alpha subunit (Gαt) which regulates its role in modulating rod photoreceptor synaptic transmission and governs it’s return to the photoreceptor outer segment after light-driven transport to the cell body and synapse. This is a well reasoned study, despite some significant errors in the presentation of the original manuscript which the authors have adequately addressed in this revised version.
